# Emergency presentation and management of acute severe asthma in children

**DOI:** 10.1186/1757-7241-17-40

**Published:** 2009-09-04

**Authors:** Knut Øymar, Thomas Halvorsen

**Affiliations:** 1 Department of Paediatrics, Stavanger University Hospital, Stavanger, Norway; 2 Department of Clinical Medicine, University of Bergen, Bergen, Norway; 3 Department of Paediatrics, Haukeland University Hospital, Bergen, Norway

## Abstract

Acute severe asthma is one of the most common medical emergency situations in childhood, and physicians caring for acutely ill children are regularly faced with this condition. In this article we present a summary of the pathophysiology as well as guidelines for the treatment of acute severe asthma in children. The cornerstones of the management of acute asthma in children are rapid administration of oxygen, inhalations with bronchodilators and systemic corticosteroids. Inhaled bronchodilators may include selective b2-agonists, adrenaline and anticholinergics. Additional treatment in selected cases may involve intravenous administration of theophylline, b2-agonists and magnesium sulphate. Both non-invasive and invasive ventilation may be options when medical treatment fails to prevent respiratory failure. It is important that relevant treatment algorithms exist, applicable to all levels of the treatment chain and reflecting local considerations and circumstances.

## Introduction

Asthma is the most common chronic disease of childhood in the western countries, and the incidence has continuously been rising during the last decades [1]. In a recently published study from Norway, the accumulated lifetime prevalence of asthma in 10 year old children was as high as 20% [2]. The majority of children with asthma have stable disease, and only a minority experience exacerbations needing hospitalisation or emergency room visits. In older children, recent advances in treatment seem to have reduced chronic morbidity as well as the number of acute exacerbations [3,4]. In infants and younger children, this goal may be more difficult to achieve, given the heterogeneity of obstructive lung disease in this age group. Viral wheeze is a very common clinical scenario in young children, and identification and proper treatment of subjects with potential for development of asthma and future exacerbations is still an unresolved challenge [5]. Furthermore, in all age groups, failure of adherence to regular anti-inflammatory treatment schemes may be an important reason why acute asthma is still a common cause of unscheduled hospitalisations in childhood. Therefore, physicians who care for acutely ill children will regularly be faced with acute severe asthma.

During recent years several guidelines have been published on treatment of stable as well as on exacerbations of asthma. Few of these guidelines have focused particularly on childhood asthma. The aim of this article is to review current knowledge of acute severe asthma in childhood, with special emphasis on the acute management.

## Methods

We performed a thorough search in PubMed with the following words in different combinations; asthma, children, severe, attack, exacerbation, epidemiology, pathophysiology, guidelines, treatment, management, oxygen, adrenaline, b2-agonist, anticholinergics, theophylline, steroids, magnesium, helium, CPAP, BiPAP, ventilation. Included studies and papers were not systematically evaluated regarding design and quality. However, we have emphasised recent guidelines, Cochrane reviews and other expert reviews.

## Clinical definitions

There is no clear definition of an asthma exacerbation [6]. However, in clinical trials it has often been defined as requirement for hospitalisation, or need for systemic corticosteroids [7,8]. Status asthmaticus may be defined as wheezing which does not respond to initial treatment with inhaled bronchodilators [9,10].

## Epidemiology

The majority of asthma exacerbations are mild or moderate and may be treated at home by the parents or by physicians outside hospitals. However, in parallel to the increase of asthma prevalence during the recent decades, the number of children hospitalised for asthma and wheezing disorders has also increased [3,4,11] Hospitalisations for asthma and wheezing disorders are most common during the first years of life; in our area ranging from 104/10000 children in the age group 1-2 years to 7/10000 in the age group 9-13 years [3], altogether constituting 16% of all emergency admissions in 2003 [12]. The hospitalisation rates for asthma in older children as well as re-admissions in all age groups seem to have declined during the last decades [11]. Some recent studies from the last few years indicate that also the overall admission rates for asthma and wheezing disorders have began to level off or even decline in Europe and the USA [8,9,12]. This development has been paralleled by an increase in the regular use of inhaled corticosteroids, suggesting that acute attacks at least partly may be a preventable complication in asthmatic children [8,11].

In preschool children, exacerbations of asthma and wheezing disorders are far more common in boys than in girls [3,8,12,13]. With increasing age, this pattern is reversed, and adult females are twice as likely to be hospitalised for asthma as adult males [7,8].

In the northern hemisphere there seems to be a seasonal pattern for asthma exacerbations in school children, with a steep rise to a peak during the first part of September from the lowest incidence during the summer months ("the September epidemic") [8]. This is probably due to an increased exposure to viral infections after school recommences. Although not so clear, a similar pattern has been observed also for pre-school children [8].

Even if severe asthma exacerbations are relatively common, mortality from asthma in children is rare and declining [8,14,15]. In the UK the mortality rate for children 0-14 years is less than one per 100.000 children per year [14]. In contrast, there has been a vast increase in the economic costs associated with asthma. However, the main economic burden of childhood asthma is linked to indirect costs, long-term follow up and medication, and not to hospitalisation [1].

## Pathophysiology

Asthma is associated with a chronic inflammation of the airway mucosa, involving a complex interaction between T-lymphocytes, neutrophils, eosinophils, epithelial cells and mast cells [9,16,17]. Cytokines and other mediators such as histamine, leukotrienes and platelet-activating factor are released from these inflammatory cells, and complex interactions between cells and mediators lead to structural and physiological changes and exposed parasympatic nerve endings [9,10,16,17]. Airway hyperreactivity is a physiological consequence of these processes, providing the asthmatic child with airways primed for a range of triggers that may lead to further airway obstruction and clinically to asthma exacerbations [9,10,16,17]. The main trigger in the paediatric age group is viral airway infections, with rhinoviruses being the most common [18]. In addition, allergens, tobacco smoke, environmental irritants, exercise, stress and gastroesophageal reflux may, separately or by concomitant action, initiate a deterioration of the chronic disease and an asthma attack (acute in chronicum) [8-10,16,18]. In some children, food allergy may trigger an acute systemic anaphylactic response, including severe airway obstruction. During an asthma attack, the chronic inflammation is aggravated by degranulation of mast cells and release of histamine, leukotrienes and other mediators, inducing mucosal vasodilatation and oedema, increased mucous secretion and smooth muscle contraction, particularly in the medium sized and small airways [10]. Thereby, the size of the airway lumen decreases resulting in increased resistance to air flow, particularly towards the end of expiration at low lung volumes. The severe airflow limitation will further lead to premature airway closure. To compensate, the patient increases end-expiratory lung volume by increasing functional residual capacity (FRC), resulting in pulmonary hyperinflation and air trapping [10]. Further, operational lung volume is shifted away from the range with the most severe expiratory airflow limitation. Consequently, airflow resistance is reduced while the work of breathing and the sense of dyspnoea are increased since the inspiratory muscles are put in a mechanically disadvantageous position [10,19].

Airway obstruction, hyperinflation and air trapping may lead to ventilation/perfusion mismatch and hypoxemia [10]. Hypoxemia and the increased work of breathing may result in anaerobic muscle work and accumulation of lactate. The metabolic acidosis may be further aggravated by dehydration from poor fluid intake. During an asthma attack, metabolic acidosis may initially be compensated for by hyperventilation and a respiratory alkalosis, but as respiratory failure develops, increasing arterial CO2 will result in a respiratory acidosis and a further decrease in arterial pH [9,10].

Increased airflow resistance and pulmonary hyperinflation combined with increased work of breathing and disturbances in the acid/base balance may impair cardiac function. During a severe asthma attack, the negative intrapleural pressure will rise, increasing left ventricular afterload and the risk of pulmonary oedema [20]. Pulmonary vasoconstriction due to hypoxemia, acidosis and increased lung volume will also increase the right ventricular afterload. Altogether, these changes may result in decreased cardiac output and decreased alveolar diffusion, further increasing both hypoxemia and acidosis [10]. Fluid overload caused by overhydration during treatment or fluid retention associated with inappropriate secretion of antidiuretic hormone, will put the patient further at risk of pulmonary oedema [21,22].

**Figure 1:**
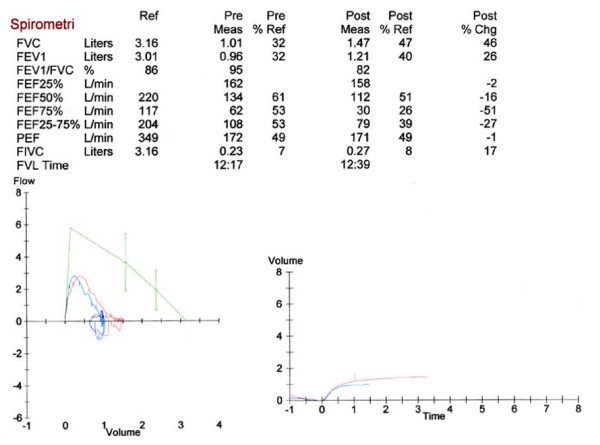
**Lung function testing in a girl with severe asthma**. Results of lung function testing of a 13 year old girl with a severe asthma exacerbation. Spirometry taken during the first day of hospitalisation measured before (blue line) and 15 minutes after (red line) inhalation with a nebulised β2-agonist (Salbutamol 1.0 mg/10 kg). Results demonstrate severely decreased lung function, and further poor reversibility probably due to long standing inflammation and downregulation of β2-receptors.

The pathophysiology of an asthma attack is influenced by the age of the patient and the trigger involved. In young children, viral aetiology with mucosal oedema predominates, and muscular bronchoconstriction is less important. Conversely, in older children, and particularly during attacks triggered by allergens, acute bronchospasm is the most important factor. These discrepancies also influence the clinical course as well as the response to treatment [23]. Asthma exacerbations mainly involving inflammatory processes may require time to develop and to resolve, and symptoms therefore tend to increase and improve relatively slowly. In these cases, airway narrowing may mainly be due to inflammatory changes, and there may be an associated down-regulation of β-receptors [24]. Consequently, the response to β2-agonists may be limited (figure 1). In contrast, allergen induced attacks may develop very rapidly with bronchoconstriction as the dominating pathophysiology, thereby also responding quickly to bronchodilator treatment.

## Assessment

### Clinical assessment

The most common symptoms in a child with acute asthma are cough, wheeze, and prolonged expiration. Objective signs include a prolonged expiratory phase, recessions, use of accessory respiratory muscles and cyanosis. On auscultation, varying degrees of high and low frequency expiratory sounds may be heard. In severe and rapid developing attacks the child may even present with respiratory failure or frank cardiopulmonary arrest.

Different grading systems have been proposed to evaluate the severity of acute asthma in children [25-27], but no firm consensus exists. A clinical grading system for bronchopulmonary obstruction has been proposed (table 1) and applied in treatment recommendations in a Nordic consensus report [25]. It is important to bear in mind that the extent of wheeze does not necessarily reflect the extent of bronchopulmonary obstruction, since some degree of airflow is required to produce a wheeze [28]. Therefore, *decreasing *wheeze and breath sounds and a "quiet chest" in a child with increasing respiratory efforts may signal imminent respiratory failure. Conversely, increasing wheeze in a child with severe asthma may indicate improvement. Development of respiratory failure is clinically best recognised by close observation of the general condition of the child, the ability to speak or cry, the mental status and level of anxiety, the skin colour and the movements of the thoracic cage and abdomen during the respiratory cycle [10]. Inability or unwillingness to lie down may be an ominous sign in a child with acute severe asthma.

**Table 1:** Symptom score by clinical assessment in children with asthma (modified from K. Aas [25]).

P0.	Normal; no signs of bronchopulmonal obstruction
P1.	No dyspnoea. Slightly faint respiratory sounds.

P2.	No dyspnoea. Moderate rhonchi. Slightly prolonged expiration. The expiration may be audible.

P3.	No dyspnoea at rest. Abundant rhonchi. Slight use of auxiliary respiratory muscles. Low grade jugular recessions may be present.

P4.	Slight dyspnoea at rest. Abundant rhonchi. Obvious use of auxiliary muscles. Jugular and intercostal recessions. No cyanosis

P5.	Severe dyspnoe at rest. Abundant rhonchi. Wheezy expiration audible without stethoscope. Jugular, intercostal and subcostal chest recessions. Slight cyanosis may be present.

P6.	Alarming obstruction., often both inspiratory and expiratory. Faint respiratory sounds. Chest recessions. Use of auxillary respiratory muscles and high respiratory rate. Cyanosis may be present but not mandatory.

Children with a special risk for severe or life-threatening asthma attacks are those with a history of frequent use of b2-agonists, frequent or recent treatment with oral corticosteroids, a previous history of severe asthma and chronic severity with impaired lung function [8].

### Laboratory assessment

A chest x-ray may be relevant in the search for underlying complications such as pneumonia or air leakages. However, in moderate asthma attacks a chest x-ray rarely leads to changes of treatment [29].

Pulse oximetry is a reliable and noninvasive measure of oxygenation and should be used in all patients to guide oxygen supplementation. However, oxygen saturation is not a good parameter of adequate ventilation in children who receives oxygen treatment. Thorough and repeated clinical assessments are required to discover imminent respiratory failure. Blood gas analyses may support the clinical judgement, as increasing levels of CO2 is an ominous sign. During a moderate asthma attack, a capillary blood gas analysis may be sufficient, while in patients admitted to an intensive care unit, arterial blood gas analyses should be routine [25]. Sequential measurements are important as respiratory alkalosis with hypocarbia is common during the early phases of an asthma attack, while normalisation and a subsequent increase in the pCO2 may be important indicators of clinical deterioration [10].

## Management

The cornerstones of acute asthma management in childhood are oxygen, inhalation of bronchodilators and systemic corticosteroids. Additional treatment should be included as required. Acute asthma is often associated with anxiety, which may further increase dyspnoea and bronchopulmonary obstruction. Reassurance is therefore important, both directly but also indirectly through the parents. The clinical value of painful procedures must be considered against their possible aggravating effects. Once established, an indwelling arterial line vastly reduces the need for subsequent painful procedures.

### Oxygen

Oxygen must be considered as a drug in a situation of acute asthma, reducing hypoxic pulmonary vasoconstriction and interfering with the ventilation-perfusion mismatch characteristic for severe bronchoconstriction [30]. Oxygen should be delivered to achieve satisfactory oxygen saturation in obstructive children with suspected or verified hypoxia. No controlled studies have evaluated which level of oxygen saturation that is adequate during an acute asthma attack, but recent guidelines recommend that oxygen saturation in children should be kept above 95% [26]. Oxygen may be delivered by a face mask or by nasal cannulae, and the dose should be adjusted by continuous monitoring by pulse oxymetry. Oxygen at a rate of 6-8 litres per minute should be used to deliver nebulised drugs [26]. In severe cases, oxygen should be administered before other drugs and before assessment is completed [26].

### Fluid

Acute asthma in children is often preceded by periods of poor fluid intake and vomiting and may therefore be associated with dehydration. Dehydration may increase metabolic acidosis, and treatment should be aimed at restoring normovolemia by oral (preferably) or by intravenous fluid substitution [10]. Overhydration will increase pulmonary oedema and must be avoided. The syndrome of inappropriate antidiuretic hormone (SIADH) has been described in severe asthma attacks, and careful monitoring of electrolyte and fluid balance is therefore important [9,10,21,22].

### Injection of adrenaline (epinephrine)

Intramuscular injection of adrenaline 10 μg/kg (0.1 ml per 10 kg body weight of adrenaline 1 mg/ml) may be given in severe bronchoconstriction during anaphylaxis. This treatment may also be an initial option in very severe exacerbations of asthma and in situations where other treatment options are not available within reasonable time [9,26].

### Inhalations with β2-agonists

There is substantial documentation for the effect of inhaled β2-agonists in acute childhood asthma [10,26,31]. The drug is traditionally nebulised, and dose recommendations for salbutamol (albuterol) vary between 0.5-1.5 mg/10 kg bodyweight, mixed in 2-5 ml NaCl 9 mg/ml [10]. Inhalations should preferably be given via a face mask, and if necessary delivered with oxygen. During initial therapy, β2-agonists are often given intermittently, as repeated inhalations every one to three hours [26]. There is, however, evidence suggesting that continuous administration of nebulised β2-agonists may have a better and prolonged bronchodilatory effect compared to intermittent therapy [9,10,31]. A sustained stimulation of β2-receptors is accomplished, and a possible rebound bronchoconstriction reported during intermittent therapy is prevented [10,31]. A recommended dose for children is 0.15 mg/kg in 5 ml NaCl 9 mg/ml given repeatedly by continuous inhalation. This has been reported to be safe and well tolerated [31]. Recent guidelines suggest a practical approach with continuous inhalation of β2-agonist during the first hour(s) of treatment and thereafter intermittent inhalations on-demand [26].

In cases with a gradually developing inflammation one should remember the possibility of a poor response to β2-agonists due to downregulation of β-receptors (figure 1) [24]. Other types of inhalations such as adrenaline and ipratropium bromid may be beneficial in such cases (se below) [31].

One should also keep in mind that β2-agonist may have stressful effects on the child, and in some cases high doses may in fact become counter-productive. Therefore, when the dose intervals are shorter than the half life of the drug, or if the strategy of continuous administration is employed, one should carefully consider and monitor the general condition of the child. An often used rule of thumb is that β2-agonist should be administered until development of significant side effects, a strategy requiring close monitoring by skilled personnel.

There are now several studies demonstrating that pressurised metered dose inhalers (pMDI) in combination with spacers are as good as or even more effective than nebulisers for intermittent administration of β2-agonist in children with moderate to severe acute asthma [31-35]. This may be the obvious choice for treatment of asthma exacerbations in children at home, and should be included in all written treatment plans. It may, however, also be used initially in emergency outpatient settings as well as in paediatric emergency wards [31]. In mild attacks, 2-4 puffs of salbutamol 0.1 mg/dose may be sufficient (0.2 - 0.4 mg), whereas in more severe attacks 10 puffs of salbutamol may be needed [31]. Oxygen cannot be given with a pMDI and spacer, excluding this method in the most severe attacks. However, in children without initial oxygen requirements, β2-agonist administered via a pMDI and spacer was less likely to provoke hypoxia and tachycardia compared to the administration via a nebuliser [32,35]. Therefore, pMDI and spacer has been recommended as the preferred mode of administration for β2-agonist in paediatric acute asthma [31].

### Nebulised adrenaline

In infants and young children with acute asthma and wheezing, bronchial smooth muscle spasm is not as prominent as in older children, and mucosal oedema and secretion may dominate the pathophysiology [36]. Therefore, inhaled β2-agonists may be less efficient. Nebulised adrenaline has a rapid but short acting effect on mucosal oedema and may be of value as initial treatment also in severely obstructed older children, before administration of inhaled β2-agonists.

Studies on the effects of nebulised adrenaline in children of different ages with bronchopulmonary obstruction reach various conclusions. Some are positive [37-40] whilst others conclude negatively [41,42]. In Nordic consensus and national protocols, nebulised adrenaline is recommended in young children (< 2 years) with acute asthma, followed by β2-agonist [25,36]. The recommended dose is racemic adrenaline 2 mg in children < 6 months of age and 4 mg in older children, inhaled in 3-5 ml NaCl 9 mg/ml [25]. Alternatively, adrenaline (1 mg/ml) may be inhaled in a dose of 1.5 mg/10 kg bodyweight (maximum 2 mg) in 2-5 ml NaCl 9 mg/ml [43].

### Inhaled anticholinergics

Current guidelines on acute paediatric asthma recommend inhaled ipratropium bromide as add-on therapy to β2-agonists. This recommendation is based on several randomised controlled trials demonstrating reduced hospital admission rates and better lung function when β2-agonists are given in combination with inhaled ipratropium bromide compared to β2-agonists given alone [44-46]. Especially when symptoms are refractory to initial treatment with β2-agonist anticholinergics should be considered [31]. The recommended dose of nebulised ipratropium bromide is (0.125-) 0.25 mg in 2-5 ml NaCl 9 mg/ml or the drug may be mixed with the β2-agonist/NaCl solution [27,31,44]. The dose may be repeated every 20 minute for the first hour and every four hour thereafter [31]. Ipratropium bromide may also be given as pMDI with a spacer at the dose of 40 μg [27].

### Steroids

An increased inflammatory response is a major part of the pathophysiology of acute asthma, and prompt treatment with corticosteroids is important. Steroids act on the pathophysiology in acute asthma in several ways, mainly by modifying the action of inflammatory cells, downregulating the release of proinflammatory cytokines and thereby controlling the airway inflammation [9,10,16,31]. Guidelines recommend that all children with moderate to severe asthma should receive systemic steroids as a part of the initial treatment [25,26]. This treatment may reduce the need for hospitalisation, reduce the risk or relapse after the initial treatment and facilitate earlier discharge from hospital [47]. There is no evidence to suggest that intravenous steroids are more effective than oral steroids, both having effect after 3-4 hours [31,48,49]. The usual recommendation for oral treatment is prednisolone 1-2 mg/kg or equivalent [31]. One study has demonstrated that a lower dose may have similar effect [50], but more studies are needed to confirm this. Intravenous hydrocortisone of 4 mg/kg or methylprednisolone 0.5 - 1.0 mg/kg every 4-6 hour are alternatives to oral steroids, but may be reserved for children unable to receive oral administration due to severity or low age [10,31].

**Figure 2:**
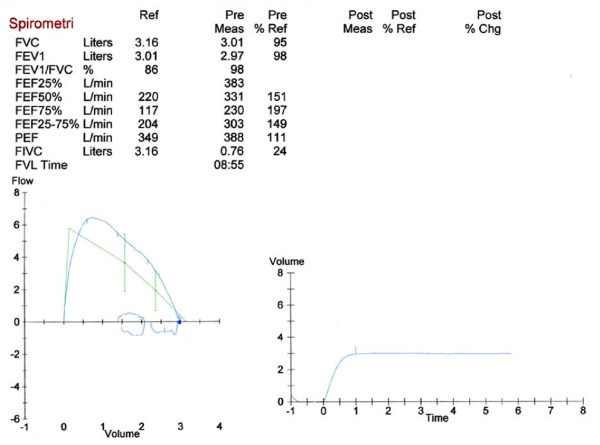
**Spirometry taken after a ten days treatment with prednisolone, approximately 1 mg/kg/day**. Green lines represent normal values.

Systemic steroids may be given repeatedly, depending on the initial response. Normally a 3-5 days course may be sufficient, but longer treatment periods may be necessary [10,26]. A prolonged course of treatment may be particularly necessary if the exacerbation is the result of longstanding untreated bronchial inflammation. Prednisolone may be given once daily, and there is no need for tapering down even after longer treatment periods [26,51]. Figure 1 demonstrates the spirometry at of a 13 year old girl at admission before and after the inhalation of nebulised salbutamol, and figure 2 the spirometry from the same girl after a 10 days course of prednisolone 1 mg/kg.

Inhaled corticosteroids are the cornerstone of regular preventive anti-inflammatory treatment of asthma, aiming at reducing chronic morbidity and preventing exacerbations [26]. It has been a widely recommended practise to double or triple the dose of inhaled steroids during exacerbations, but the data to support this is missing [31]. However, recent studies have suggested that high doses if inhaled steroids during the early phase of an asthma exacerbation may be beneficial [52,53], but this approach is not incorporated in current guidelines and more studies are needed to evaluate this issue [26].

### Additional medication

Theophylline. The positive effect from theophylline infusion on acute asthma is well documented, as are the potential for side effects and severe or even fatal complications [10,54-57]. In light of the highly efficient inhaled bronchodilators and systemic corticosteroids, a theophylline infusion therefore has no place in the routine treatment of children with asthma exacerbations [26]. In our department, theophylline given rectally or as an infusion was used in 85% of admissions for childhood asthma in 1984/1985, and in 3% in 1999/2000 [3]. However, in one study, theophylline infusion had some additional effect in children with near-fatal asthma, already receiving an aggressive regimen with multiple inhaled bronchodilators and intravenous corticosteroids [54]. Wheeler et al concluded that theophylline infusion was superior to terbutaline as add on treatment in children with status asthmaticus [56]. Theophylline may therefore be considered in children with a poor response to other treatment measures.

Intravenous b2-agonists may also be considered in children with severe asthma who do not respond to other treatments [31,58,59]. Inhaled drugs may have limited effect in children with nearly complete airway obstruction and have practical limitations in ventilated patients. Intravenous terbutaline has been shown to improve pulmonary function and gas exchange in children with status asthmaticus [31,59], whereas others have failed to demonstrate efficacy [60]. A suggested dose may be terbutaline 5-10 ug/kg/h [25], but the dose may be titrated higher [58]. However, one should bear in mind cardiac side effects such as dysrythmias, tachycardia and hypertension. Severe hypokalemia induced by β2-agonists may also aggravate possible dysrythmias [61]. The effect of inhaled β2-agonists observed in most cases, limit the need for intravenous administration to very few children [26].

Magnesium sulphate. The potential benefit of magnesium sulphate during acute asthma may be via smooth muscle relaxation secondary to inhibition of calcium uptake. Several studies have evaluated inhaled and intravenous administration of magnesium sulphate in severe childhood asthma, but results are diverging [62,63]. A recent meta-analysis, however, suggested that intravenous magnesium sulphate may be effective in children with severe acute asthma, whereas more studies are needed to evaluate the effect of inhaled magnesium sulphate [63]. The recent GINA-guidelines suggest that intravenous magnesium may be considered in acute moderate and severe asthma with incomplete response to initial treatment during the first 1-2 hours [26]. It is interesting that this treatment option is listed before intravenous theophylline. The dose of intravenous magnesium sulphate children used in studies is 25 - 100 mg/kg given over 20 minutes [10,63]. Intravenous magnesium sulphate is not studied in young children and is not included in recent guidelines for children younger than five years of age [27].

At present there is no evidence to support the use of helium oxygen therapy or leukotriene modifiers in the treatment of children with acute asthma [9,26,64,65]. Furthermore, it is important to avoid the use of sedatives because of the depressant effect on the respiratory efforts [26]. In severely agitated children one must consider the possibility of side effects and drug overdoses, particularly from adrenergic inhalation or from theophylline. In children receiving massive treatment with inhaled and/or intravenous adrenergic and/or anticholinergic drugs and maybe also intravenous theophylline, one must observe for cardiac side effects and if suspected, institute adequate measures.

### Non-invasive and invasive ventilation

A detailed presentation of the principles of non-invasive and invasive ventilation of children with severe bronchopulmonary obstruction is beyond the scope of this review. However, studies during recent years suggest that bilevel positive airway pressure (BiPAP) in children with severe asthma may improve symptoms and ventilation without significant adverse events and reduce the need for intubation and mechanical ventilation [9,65-68]. This treatment may therefore be considered in children not responding properly to initial treatment and with threatening respiratory failure. However, in younger children, lack of cooperation, stress and agitation may induce pressure leaks and prevent its use. BiPAP is contraindicated in the patient with altered mental status [65].

Intubation and positive pressure ventilation of an asthmatic child may increase bronchoconstriction, increase the risk of airway leakage and has disadvantageous effects on circulation and cardiac output [10,69]. Therefore, intubation should be avoided unless respiratory failure is imminent despite adequate institution of all available treatment measures. Absolute indications for intubation include severe hypoxia, cardiopulmonary arrest, and severe deterioration of the mental status of the child. Relative indications are progress of respiratory failure and/or increasing CO_2 _despite adequate utilisation of all available treatment measures. However, children should not be intubated based on blood gas analyses alone [9,10]. The clinical signs indicating a severe obstruction or a deteriorating clinical situation are described previously under the heading "assessment", and the importance of close observation of these signs by an experienced staff cannot be overestimated.

Before intubation, the child should be properly preoxygenated. Atropine may be indicated together with a sedative and a rapid muscle relaxant. Ketamine (1-2 mg/kg i.v) is often recommended due to its bronchodilating effect [10]. Shortly after intubation, complications such as hypotension, cardiac arrest, pneumothorax and hypoxia may develop [10,70]. Hypotension may be caused by hyperinflation with decreased veneous return to the heart, aggravated by the vasodilatory effects of medications used during intubation. Hypotension may be prevented by a fluid bolus given prior to intubation, or aggressively treated if occurring [10].

During mechanical ventilation the child should be well sedated. Ventilator setting should aim at avoiding hyperinflation and intrinsic positive end expiratory pressure (PEEP). Normally the settings will involve a low inspiratory to expiratory ratio, a low respiratory rate and low tidal volumes. Pressure control, pressure support and permissive hypercapnia may prevent air-leakage [10]. Positive end-expiratory pressure is debated [68,71].

## Management plan

**Figure 3:**
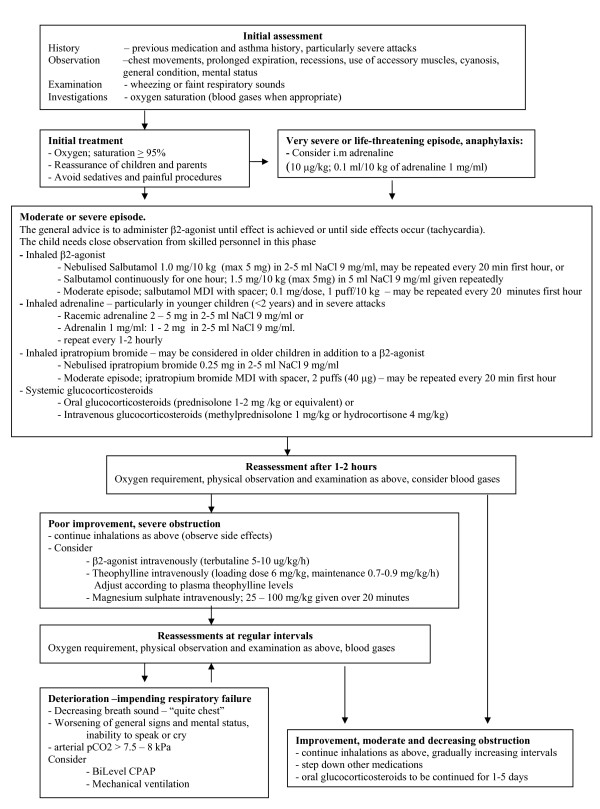
**Treatment algorithm for children with moderate or severe asthma exacerbations**.

Based on the above considerations and recent guidelines, we suggest a treatment algorithm for acute asthma in children, including dose recommendations (Figure 3). The suggested use of nebulised adrenaline has some support from the literature, but has not been included in other guidelines, for instance the GINA.

All institutions caring for children with acute asthma should provide to their staff a clear in-house treatment algorithm, taking local considerations and circumstances into account.

## Differential diagnostic considerations

Physicians facing a child with a suspected acute asthma attack should consider possible alternative diagnoses [72]. Respiratory distress resembling an acute attack of asthma can be caused by other pulmonary conditions, such as pneumonia or spontaneous pneumothorax, or by obstruction in central bronchi, such as aspiration of a foreign body, or by obstruction in the trachea or larynx, such as pseudocroup or vocal cord dysfunction. Hyperventilation may mimic as well as complicate an asthma attack, particularly in older children [72].

## Conclusion

Despite recent progress in the treatment of chronic asthma in childhood, acute exacerbations will continue to occur. Physicians working within the field of paediatric emergency medicine will therefore continue to be exposed to this clinical scenario. The cornerstones of acute asthma management in childhood are rapid onset of oxygen treatment, inhalation of bronchodilators and systemic corticosteroids. It is important that relevant treatment algorithms exist, applicable to all levels of the treatment chain and reflecting local considerations and circumstances.

## Competing interests

The authors declare that they have no competing interests.

## Authors' contributions

KØ performed a search of the literature and drafted the manuscript. TH participated in writing and evaluating the manuscript. Both authors read and approved the final manuscript.
